# Complement therapy in atypical haemolytic uraemic syndrome (aHUS)

**DOI:** 10.1016/j.molimm.2013.05.224

**Published:** 2013-12-15

**Authors:** Edwin K.S. Wong, Tim H.J. Goodship, David Kavanagh

**Affiliations:** The Institute of Genetic Medicine, Newcastle University, Newcastle upon Tyne, UK

**Keywords:** Eculizumab, Haemolytic Uraemic Syndrome, Treatment, Complement

## Abstract

Central to the pathogenesis of atypical haemolytic uraemic syndrome (aHUS) is over-activation of the alternative pathway of complement. Inherited defects in complement genes and autoantibodies against complement regulatory proteins have been described. The use of plasma exchange to replace non-functioning complement regulators and hyper-functional complement components in addition to the removal of CFH-autoantibodies made this the ‘gold-standard’ for management of aHUS. In the last 4 years the introduction of the complement inhibitor Eculizumab has revolutionised the management of aHUS. In this review we shall discuss the available literature on treatment strategies to date.

## 1 Introduction

Atypical HUS (aHUS) is the prototypical disease of complement over activation (Kavanagh et al., 2008a). Thrombocytopenia, microangiopathic haemolytic anaemia and acute renal failure are the hallmarks of haemolytic uraemic syndrome (HUS). Atypical HUS is the term used to classify any HUS not due to Shiga toxin (Stx)-producing bacteria, typically *Escherichia coli* O157:H7 (Besbas et al., 2006).

The discovery of mutations in the complement system in aHUS by Warwicker et al. (1998) was to set in train the research which was to ultimately result in the successful use of the complement inhibitor Eculizumab in aHUS.

## 2 The complement system

Complement is an ancient pathway that sits at the nexus of the immune system (Ricklin et al., 2010): protecting against invading pathogens; bridging innate and adaptive immunity (Kemper and Atkinson, 2007); and disposing of immune complexes and injured tissues and cells (Richards et al., 2007b).

**Fig. 1:**
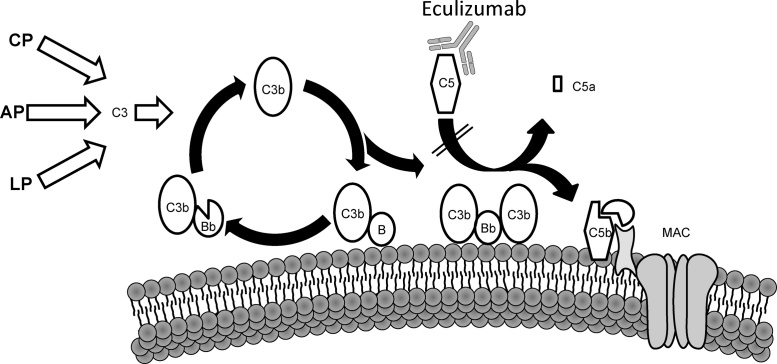
Complement activation and the mechanism of action of Eculizumab. The AP constantly undergoes ‘tick-over’ but can also be primed by the CP and LP pathways. The C3b that is formed interacts with factor B (B), which is then cleaved by factor D to form the AP C3 convertase (C3bBb). This enzyme complex is attached to the target covalently via C3b while Bb is the catalytic serine protease subunit. Because C3 is the substrate for this convertase, a powerful feedback loop is created. Unchecked, this will lead to activation of the terminal complement pathway with generation of the effector molecules; the anaphylatoxin C5a and the membrane attack complex (MAC). Eculizumab binds C5 and prevents its entry into the C5 convertase (C3bBbC3b), thus precluding cleavage into the effector molecules, C5a and C5b and ultimately the MAC.

Complement activation is mediated via different initiating triggers. The classical pathway (CP) can be initiated via IgM and IgG as well as the pattern recognition molecule (PRM) C1q. In the lectin pathway (LP) the PRMs, mannose binding lectin (MBL) and ficolins bind carbohydrates to trigger complement activation. The alternative pathway (AP) constantly “ticks over” depositing C3b on surfaces which is inactivated on host cells and amplified on foreign cells. Properdin can also bind to foreign and apoptotic cells to propagate the AP. The AP is also recruited by C3 convertases formed by the CP and LP and as such, it serves as an amplification step accounting for ∼80% of all complement activation regardless of the initial trigger (Harboe and Mollnes, 2008). All pathways subsequently converge to produce the common terminal pathway effector molecules (Ricklin et al., 2010) (Fig. 1).

The runaway complement activation of the AP has evolved to rapidly destroy invading microorganisms but to prevent collateral damage to host tissue, fluid phase (e.g. complement factor H (CFH) and complement factor I (CFI)) and membrane bound (e.g. membrane cofactor protein (MCP)) complement regulatory proteins are present. It is an imbalance between this activation and regulation on the glomerular vasculature which underlies the pathogenesis of aHUS.

## 3 The role of complement in aHUS

The last 15 years has seen the elucidation of the critical pathways involved in the pathogenesis of aHUS. Loss of function mutations in complement regulatory proteins and gain of function mutations in complement components have been described in aHUS. Similarly, autoantibodies to complement regulatory proteins have been described.

### 3.1 Complement factor H

CFH is the critical fluid-phase regulator of the AP acting via its N-terminal domains (CCPs 1–4) (Richards et al., 2007b). CFH can also protect host surfaces by binding to polyanions such as the glycosaminoglycans (GAG) of endothelial cells and exposed basement membranes (Meri and Pangburn, 1994, Schmidt et al., 2008). CFH has two GAG binding domains in CCPs 6–8 and CCPs 19–20 which have different sulphate specificities. CCPs 6–8 are predominantly responsible for binding in the eye while the C-terminal domains (CCPs 19–20) account for kidney binding (Clark et al., 2013). Additionally CFH also binds to the lipid peroxidation product malondialdehyde (Weismann et al., 2011), the acute phase proteins, C-reactive protein (Hakobyan et al., 2008, Laine et al., 2007, Sjoberg et al., 2007) and pentraxin 3 (Kopp et al., 2012) as well as necrotic cells (Sjoberg et al., 2007).

Mutations in *CFH* were first described in 1998 (Warwicker et al., 1998) and mutations in this gene are the most common genetic predisposition to aHUS, accounting for around 25% of all cases (Caprioli et al., 2001, Dragon-Durey et al., 2004, Fan et al., 2013, Geerdink et al., 2012, Maga et al., 2010, Neumann et al., 2003, Perez-Caballero et al., 2001, Richards et al., 2001, Warwicker et al., 1998). The majority of mutations in *CFH* are located in CCPs 19–20 and do not usually result in a quantitative deficiency. These C-terminal mutations fail to bind to cell surfaces and result in ineffective control of complement activation on the glomerular vasculature (Abarrategui-Garrido et al., 2008, Ferreira et al., 2009, Vaziri-Sani et al., 2006). C-terminal CFH mutants have also been demonstrated to have reduced binding to platelets resulting in increased complement activation with consequent platelet activation, aggregation and release of tissue factor-expressing micro-particles (Stahl et al., 2008). Although clustering in the C-terminal, mutations are reported throughout the molecule. N-terminal mutations in CFH (CCPs 1–4) result in ineffective control of the AP both in the fluid phase and on cell surface (Pechtl et al., 2011). The functional effects of normally secreted genetic variants in other regions of the protein remain to be determined (Kavanagh and Anderson, 2012, Tortajada et al., 2012).

CFH and the five factor H-related proteins, which arose from several large genomic duplications, reside in the RCA cluster. This homology predisposes to gene conversions and genomic rearrangements through non-allelic homologous recombination (NAHR) and microhomology-mediated end joining (MMEJ). The *CFH* mutations S1191L, V1197A, and combined S1191L/V1197A arose through gene conversion between *CFHR1* and *CFH* (Heinen et al., 2006). A hybrid (fusion) gene comprising the 21 N-terminal exons of *CFH* and the 2 C-terminal exons of *CFHR1* has been demonstrated to have arisen through NAHR resulting in aHUS (Venables et al., 2006). More recently a hybrid gene consisting of the 22 N-terminal exons of *CFH* and the 5 C-terminal domains of *CFHR3* arising through MMEJ has been reported in aHUS (Francis et al., 2012). As with C-terminal point mutations in *CFH*, these hybrid genes result in loss of cell surface complement regulation.

### 3.2 Complement factor I

CFI is a serum serine protease, which functions as a critical mediator of complement regulation by cleaving C3b and C4b in the presence of its cofactors (CFH for C3b; C4BP for C4b; MCP and CR1 for both). It is predominantly synthesised by the liver. Mutations in *CFI* account for between 5 and 10% of aHUS (Caprioli et al., 2006, Fremeaux-Bacchi et al., 2005, Kavanagh et al., 2005, Kavanagh et al., 2008b, Maga et al., 2010, Nilsson et al., 2010, Nilsson et al., 2007, Sullivan et al., 2010, Westra et al., 2010). The *CFI* mutations described in aHUS are all heterozygous. These mutations cluster in the serine protease domain and the majority result in a non-secreted protein. Functional analysis has been undertaken for a number of mutants and demonstrates a loss of both AP and CP regulatory activity in the fluid phase and on cell surfaces (Kavanagh et al., 2008b, Nilsson et al., 2010).

### 3.3 Membrane cofactor protein

MCP is a surface bound complement regulatory protein which acts as a cofactor for the CFI mediated cleavage of C3b and C4b that are deposited on host cells (Richards et al., 2007a). Mutations in *MCP* are found in around 10% of patients with aHUS (Caprioli et al., 2006, Fremeaux-Bacchi et al., 2006, Maga et al., 2010, Richards et al., 2007a, Richards et al., 2003, Westra et al., 2010). Most mutations described in aHUS reside in the extracellular 4 CCP domains that are responsible for C3b and C4b binding. Most *MCP* mutations described to date result in a quantitative defect in MCP (∼75%) (Richards et al., 2007a). The remaining mutations have been demonstrated to result in a secreted, non-functional protein (Richards et al., 2007a).

### 3.4 Activating mutations

In addition to loss of function mutations in complement regulatory proteins, gain of function mutations have been described in the complement components *C3* and factor B (*CFB*). C3 is cleaved to form the anaphylatoxin C3a and C3b, which is highly reactive, and can bind to cell surfaces via its reactive thioester. C3b can then interact with CFB in the presence of factor D to form the AP C3 convertase (C3bBb), which cleaves further C3, introducing a positive-amplification loop (Fig. 1).

Gain of function mutations in *CFB* are rare (Fan et al., 2013, Geerdink et al., 2012, Goicoechea de Jorge et al., 2007, Kavanagh et al., 2006, Noris et al., 2010, Roumenina et al., 2009, Tawadrous et al., 2010). The mutations have been demonstrated to either enhance formation of the C3bB proenzyme or form a C3 convertase more resistant to decay by the complement regulators decay accelerating factor (DAF; CD55) and CFH (Goicoechea de Jorge et al., 2007, Roumenina et al., 2009). Ultimately these mutations result in increased complement deposition on endothelial cells (Roumenina et al., 2009).

Mutations in *C3* appear to be more common occurring in 2–10% of aHUS (Fan et al., 2013, Fremeaux-Bacchi et al., 2013, Fremeaux-Bacchi et al., 2008, Geerdink et al., 2012, Lhotta et al., 2009, Maga et al., 2010, Noris et al., 2010, Roumenina et al., 2012, Sartz et al., 2012). As with *CFB* mutations, the C3 mutants either have increased resistance to regulation or bind to CFB with higher affinity resulting in increased C3 convertase formation (Roumenina et al., 2012, Sartz et al., 2012). These mutations result in increased complement activation on platelets (Sartz et al., 2012) and glomerular endothelium (Roumenina et al., 2012)

### 3.5 Acquired complement abnormalities in aHUS

As well as the genetic abnormalities described in aHUS, autoantibodies to CFH have also been linked to disease in 4–14% of aHUS patients (Abarrategui-Garrido et al., 2009, Dragon-Durey et al., 2005, Foltyn Zadura et al., 2012, Jozsi et al., 2007, Maga et al., 2010, Moore et al., 2010, Noris et al., 2010). In cohorts of paediatric patients, this figure is as high at 25% (Hofer et al., 2012). Most of the reported studies suggest that the anti-CFH Abs bind predominantly to the C-terminus (Dragon-Durey et al., 2005, Jozsi et al., 2007, Moore et al., 2010) although in some cases there is a polyclonal response (Blanc et al., 2012). Cross reactivity of the anti-CFH Ab has also been seen to CFHR1 (Blanc et al., 2012, Moore et al., 2010, Strobel et al., 2011) and CFHR2 (Blanc et al., 2012). Several studies have demonstrated various functional consequences of anti-CFH Abs. The antibodies have been demonstrated to reduce binding to C3b (Blanc et al., 2012, Jozsi et al., 2007). They perturb CFH-mediated cell surface protection and in some individuals the autoantibodies also impair cofactor activity (Blanc et al., 2012) or decay accelerating activity (Dragon-Durey et al., 2004). These functional studies suggest a pathogenic role for CFH autoantibodies in aHUS.

Autoantibodies to CFI are much rarer than anti-CFH Abs (0–2%) (Foltyn Zadura et al., 2012, Kavanagh et al., 2012). Anti-CFI Abs were seen to form immune complexes in serum however functional analysis revealed only a minor effect on fluid phase co-factor activity (Kavanagh et al., 2012). The co-existence of functionally significant mutants in the majority of patients, added to the lack of correlation of anti-CFI Ab titre and disease activity raise the possibility that they are an epiphenomenon rather than a direct cause of disease.

## 4 Incomplete penetrance

Incomplete penetrance has been reported for all the genes associated with aHUS. Penetrance has been reported at around 50% for individuals carrying *CFH*, *CFI*, *MCP*, and *CFB* mutations (Caprioli et al., 2003, Kavanagh and Goodship, 2010, Sullivan et al., 2011) and slightly lower for *C3* mutations, albeit with small numbers (Lhotta et al., 2009). This suggests that the penetrance is altered by other environmental and genetic modifiers.

It is increasingly recognised that patients may have mutations in more than one complement gene (Bienaime et al., 2010, Cruzado et al., 2009, Esparza-Gordillo et al., 2006, Maga et al., 2010, Sellier-Leclerc et al., 2007) or mutations in one complement gene in addition to autoantibodies to complement regulators (Kavanagh et al., 2012, Moore et al., 2010). In a study of 795 aHUS patients the European Working Party on Complement Genetics demonstrated that at least 3.4% of aHUS cases will have more than one mutation. 8–10% of patients with mutations in *CFH*, *C3* or *CFB* had combined mutations whereas 25% of patients with mutations in *CFI* or *MCP* had combined mutations (Bresin et al., 2013). The penetrance increased as the number of mutations in a patient increased (Bresin et al., 2013).

In addition to mutations in complement genes a number of single nucleotide polymorphisms (SNPs) in *CFH* have been demonstrated to be associated with aHUS in several studies (Abarrategui-Garrido et al., 2009, Caprioli et al., 2003, Ermini et al., 2012, Esparza-Gordillo et al., 2005, Fremeaux-Bacchi et al., 2005, Pickering et al., 2007). A haplotype in *CFH* (*CFH*-H3; tgtgt) composed of these SNPs increases this risk of aHUS 2–4-fold (Fremeaux-Bacchi et al., 2013, Pickering et al., 2007). A haplotype block in *MCP* (*MCP*ggaac) comprising 2 SNPs in the promoter region has been associated with a 2–3 fold increased risk of aHUS (Esparza-Gordillo et al., 2005, Fremeaux-Bacchi et al., 2013, Fremeaux-Bacchi et al., 2005). Some of these studies have suggested that this risk occurs exclusively in those patients already carrying complement mutations (Ermini et al., 2012, Esparza-Gordillo et al., 2005). A SNP in C4b binding protein (R240H) was associated with aHUS in cohorts from the UK and France but could not be replicated in a Spanish cohort (Blom et al., 2008, Martinez-Barricarte et al., 2009). In a study examining SNPs in 47 complement genes in 2 separate cohorts, SNPs in *CFHR2* and *CFHR4* were also associated with aHUS. In this study there were no reproducible associations between SNPs and aHUS outside the RCA cluster (Ermini et al., 2012).

Thus, haplotypes and SNPs act together with mutations and inhibitory autoantibodies to increase the penetrance of disease. However even when a patient has multiple genetic risk factors, disease may not present until middle age suggesting a triggering stimuli is required for disease to manifest. In individuals with mutations, these stimuli have been suggested to be upper respiratory tract infections, fevers, pregnancy, drugs and non *Escherichia coli* diarrhoeal illnesses as potential triggers (Bento et al., 2010, Caprioli et al., 2006, Edey et al., 2008, Fakhouri et al., 2010, Fremeaux-Bacchi et al., 2013, Goodship and Kavanagh, 2010, Noris et al., 2010). It is likely that these events trigger the AP which susceptible individuals are unable to adequately control, resulting in aHUS.

## 5 Diacylglycerol kinase *ɛ* and HUS

In addition to complement mediated aHUS (Lemaire et al., 2013) have recently demonstrated that homozygous or compound heterozygous mutations in diacylglycerol kinase ɛ (*DGKɛ*) cause disease. The clinical phenotype of these patients appears distinct from complement mediated aHUS. All individuals presented with aHUS before one year (mean 0.5 years, range 0.3–0.9 years). In those recovering from the acute episode of aHUS, microscopic haematuria and proteinuria persisted and progression to CKD was common. In keeping with this distinct phenotype, a recessively inherited MPGN like illness with proteinuria and renal failure has also been linked to *DGKɛ* (Ozaltin et al., 2013). In contrast to serum complement mediated aHUS, recurrence in renal transplants was not seen.

## 6 Diagnosing aHUS

Having diagnosed a thrombotic microangiopathy (TMA), the initial management involves differentiating between thrombotic thrombocytopenic purpura (TTP), Stx-HUS, and aHUS (reviewed Loirat and Fremeaux-Bacchi, 2011). Rapid exclusion by microbiological analysis for Stx-producing *E. coli* and analysis of ADAMTS13 activity can lead to a diagnosis of aHUS. Following exclusion of Stx-HUS and TTP, precipitating events and the underlying genetic defects predisposing to aHUS should be sought.

Prior to initiation of plasma exchange (PE) serum should be obtained for levels of C3, C4, CFH and CFI, and a complement antibody screen. FACS analysis of peripheral blood mononuclear cells for MCP expression should be performed. Genetic testing including a method to detect copy number variation should be undertaken.

## 7 Prognosis

Historically, the overall prognosis for patients with aHUS has been poor with up to 48% of children and 67% of adults dying or reaching end-stage renal disease (ESRD) within 5 years (Fremeaux-Bacchi et al., 2013, Noris et al., 2010). The outlook is predicted by the genotype with *MCP* mutations carrying the best prognosis. In several large cohorts, no patient with an MCP mutation died at first presentation and at 5 years only 35% had reached ESRD. Mutations in *CFH*, *CFI* or *C3* all carry poor outcomes. At 3–5 years follow up, up to 77% of patients with *CFH* mutations had developed ESRD or had died. Only 30–40% of individuals with *CFI* and *C3* mutations will be alive with native kidney function at 3–5 years (Fremeaux-Bacchi et al., 2013, Noris et al., 2010). The prognosis of aHUS with *CFB* mutations is also poor (Goicoechea de Jorge et al., 2007, Noris et al., 2010, Roumenina et al., 2009).

In addition to predicting the outcome in native kidneys, the outcome following renal transplantation is determined largely by the underlying genetic abnormality. Graft failure is predominantly due to aHUS recurrence which occurs in 60–70% of patients (Bresin et al., 2006, Le Quintrec et al., 2013). In individuals with mutations in *CFH* the recurrence rate is >80%. Similarly activating mutations in *C3* and *CFB* also have a high risk of renal recurrence. Initial studies all suggested that mutations in *CFI* carried a poor prognosis although more recently one study failed to replicate this data (Le Quintrec et al., 2013). Unlike the serum complement proteins, the recurrence rate in individuals with mutations in *MCP* is very low (Loirat and Fremeaux-Bacchi, 2008). As MCP is a membrane regulator, a renal allograft will correct the complement defect and protect against aHUS.

## 8 Treatment

### 8.1 Plasma exchange

Until the introduction of Eculizumab, PE has been considered the ‘gold-standard’ for management of aHUS. The replacement of non-functioning complement regulators and hyper-functional complement components (e.g. gain of function mutations) in addition to the removal of CFH-autoantibodies made PE a logical choice (reviewed in European (Ariceta et al., 2009) and UK (Taylor et al., 2010) guidelines on aHUS treatment). The consensus based guidelines recommended that PE should be commenced as soon as possible on diagnosis of aHUS and performed daily with dose titration to control haemolysis. Once haemolysis has been controlled, PE can be slowly withdrawn, although individuals with genetic defects in the complement system are frequently plasma dependent and require long term plasma therapy (weekly/biweekly) to maintain remission. In adults, only once ADAMTS13 deficiency is excluded should Eculizumab be considered.

## 9 Eculizumab

### 9.1 Pharmacology

Eculizumab is a recombinant, monoclonal antibody directed against human complement component C5 (Rother et al., 2007). Molecular modelling has suggested that Eculizumab binds C5 and prevents its entry into the C5 convertase, thus precluding cleavage into the effector molecules, C5a and C5b (Zuber et al., 2012a) (Fig. 1).

Eculizumab has been humanized, replacing the murine heavy-chain constant region with a human hybrid IgG2/IgG4 constant region (Thomas et al., 1996). This hybrid region utilises the desirable properties both of IgG2, which fails to bind Fc receptors (Canfield and Morrison, 1991), and IgG4, which does not activate complement (Tao et al., 1993), to reduce the pro-inflammatory potential of the antibody.

Eculizumab is administered by intravenous infusion and has a half-life of ∼11 days (Rother et al., 2007). Complete blockade of the terminal pathway of complement occurs in vivo with serum concentrations above 35 μg/mL (Park et al., 2011). Human tissue cross-reactivity has not been seen in Eculizumab binding studies (Rother et al., 2007) and little transplacental transfer of Eculizumab has been reported in pregnant women with paroxysmal nocturnal haemoglobinuria (PNH) on Eculizumab (Kelly et al., 2010, Luzzatto et al., 2010).

### 9.2 Evidence in animal models for the use of Eculizumab in aHUS

Pickering et al. (2007) generated a transgenic mouse lacking the C-terminal domains of factor H (Cfh−/−Δ16–20). Without the GAG binding domains, endothelial cell complement regulation was lacking and the mice spontaneously developed aHUS. Goicochea de Jorge et al. (2008) subsequently crossed the Cfh−/−Δ16–20 with a C5-deficient mouse which did not develop aHUS, suggesting a critical role downstream of C3b generation in aHUS and thus providing a rationale for the use of Eculizumab in human disease.

### 9.3 The use of Eculizumab in aHUS

The use of Eculizumab in aHUS was first reported by Gruppo and Rother (2009) and Nurnberger et al. (2009) as two separate cases published in the New England Journal of Medicine in 2009. In this review, we describe the published experience of the use of Eculizumab in the treatment of aHUS. In total 44 cases have been summarised in Table 1, Table 2, Table 3. These are limited to individual case reports and series. Data from prospective clinical trials of Eculizumab in the treatment of aHUS awaits publication.

**Table 1:** Summary of 18 patients receiving Eculizumab for the treatment of aHUS in the native kidney.

Reference	Mutation	Age at onset of aHUS	Response to PE	Time from aHUS diagnosis to Ecu	Response to PE at time of Ecu	SCr (μmol/L) at time of Ecu	Achieved TMA remission	Ecu dosing	Evolution of aHUS last SCr (μmol/L)	Follow-up
Gruppo and Rother (2009)	NI	<8d	PI sensitive 3 relapses over 11m	19m	Resistant	265	Y	Ongoing	Remission 35	2y 4m
Fremont et al. (2009)	*CFH*	4y	PE partially sensitive	Months	Partially sensitive	80	Y	Ongoing	Remission 26	10w
Mache et al. (2009)	NI	17.8m	PE sensitive 3 relapses at PE taper	3m	Resistant	Dialysis	Y	Single dose	Relapse 2 weeks further 3 doses ESRD	Ecu discontinued
Kose et al. (2010)	NI	18y	NK	Months	Resistant	∼300	Y	Single dose	Relapse at 2m ESRD	NA
Lapeyraque et al. (2011)	*CFH* S1191L V1197A	7m	PE/PI sensitive 11 relapses over 5 y	Years	Resistant^b^	108	Y	Ongoing	Remission 44	1y 3m
Prescott et al. (2010)	*CFI* A258T	47y	PE sensitive relapse 2w after PE cessation	18d	Resistant	610	Y	Ongoing	Remission 230	7m
Ohanian et al., 2011a, Ohanian et al., 2011b	NI	50y	NA^a^	6d	No PE^a^	600	Y	Ongoing	Remission 125	6m
Tschumi et al. (2011)	*CFH* C611Y	9y	2 relapses during PE taper	126d	Sensitive^c^	220	Y^e^	Ongoing	Remission ∼100	2y
Ariceta et al. (2012)	NI	28d	Resistant to 4× PI Intolerant of PE	11d	Resistant^b^	Dialysis	Y	Ongoing	Remission normal SCr	14m
Carr and Cataland (2012)	*CFH*	20y	Resistant	<2w	Resistant	Dialysis	Y	9m	Relapse 6m later Remission again with Ecu	NK
Cayci et al. (2012)	*CFI* K434R	10y	10 sessions of PE	<2w	Resistant	Dialysis	Y	3× doses	Remission ∼44	4m
Garjau et al. (2012)	*MCP* c.286 + 1G > C	44y	PE for 90 days	90d	Resistant	Dialysis	Y	27w	ESRD at 27w	Ecu discontinued
Kim et al. (2012)	*CFH* Y1190H	7m	3 relapses despite PE	4m	Resistant	Dialysis	Y.	Ongoing	Remission 75	18m
Dorresteijn et al. (2012)	NI	6y	Initial response to PE, relapse on PI	11w	Resistant	Dialysis	Y	Ongoing	Remission 80	9m
Giordano et al. (2012)	*CFH* c3514G > T	1y	21 PI	3m	Resistant	Dialysis	Y	Ongoing	Remission 44	12m
Vilalta et al. (2012)	*CFH* D1119N	1y	PE sensitive	5m	No PE^d^	Dialysis	Y	Single dose	Relapse after 8w restarted on Ecu remission 26.5	2.5y
De et al. (2010) and Heinen et al. (2013)	*CFH* S1191L	6m	PE dependent	11y	Sensitive	Dialysis	Y	Ongoing	ESRD Ecu continued to maintain TMA remission	NK
Povey et al. (2013)	NK	NK	PE resistant	Months	Resistant	Dialysis	Y	Ongoing	Remission near-normal SCr	NK
Gilbert et al. (2013)	*CFB* K323Q	4m	NA^a^	7 days	No PE^a^	20	Y	Ongoing	Remission 12	6m

NI: not identified; NK: not known; NA: not applicable; SCr serum creatinine; Ecu: Eculizumab; PI plasma infusion; PE plasma exchange.

a Commenced on Ecu first line.

b Was receiving plasma infusion.

c Stopped due to allergic reaction.

d PE previously stopped due to intolerance.

e Already in remission when Ecu commenced.

**Table 2:** Summary of 15 patients receiving Eculizumab for the treatment of aHUS recurrence following renal transplantation.

Reference	Mutation	Previous transplants	Age and post-Tx course	Time from recurrence to Ecu	SCr (μmol/L) at time of Ecu	Eculizumab dosing	TMA remission achieved	Recurrence if Ecu stopped	Outcome Ecu continued SCr (μmol/L)	Follow up
Nurnberger et al. (2009)	*CFH* Y475S	1st Tx recurrence at 5w, PE resistant, graft loss	37y 2nd Tx, recurrence at 6w. PE resistant	5d	132	Single dose	Y	Likely (21m) graft loss	NA	NA
Chatelet et al. (2010)	*C3* R570Q	1st Tx, recurrence at 5m Graft loss at 2y.	43y 2nd Tx, recurrence at 3y, PE dependent	15m	320	Ongoing	Y	NA	2 recurrences of TMA^b^ 230	2y 5m
Legault and Boelkins (2009)	ND	No	34y 1st Tx recurrence at 1m and 5m. PE sensitive then resistant	9m	323	Ongoing	Y	NA	Remission 238	6m
Davin et al. (2010)	*CFH* S1191L	1st Tx, recurrence at 3d, graft loss. 2nd Tx under PE, recurrence at 10w graft loss	17y 3rd Tx, prophylactic PE. Recurrence at 4m. rescue PE intolerant at 10m.	10m	131	Ongoing	Y^a^	NA	Remission 130	1y 10m
Larrea et al. (2010) and Loirat and Fremeaux-Bacchi (2011)	NI	No	22y 1st Tx recurrence at 12d PE resistant	9d	415	Single dose	Y	Recurrence (11.5m) Ecu resumed	Subsequent humoral rejection Ecu stopped and graft loss	NA
Zuber et al. (2011)	*CFH*	1st Tx, recurrence, graft loss	24y 2nd Tx, prophylactic PI/PE recurrence 1d PE resistant	4d	500	Ongoing	Y	NA	Remission 62	9m
Al-Akash et al. (2011)	*C3* R570W	1st Tx, recurrence at 4y, graft loss 2nd Tx recurrence at 2m graft loss	15y 3rd Tx, prophylactic PE, recurrence at 2m, PE partially sensitive	∼20d	202	Ongoing	Y	NA	Remission 115	1y 5m
Duran et al. (2012)	*CFH* Q1172X	No	32y 1st Tx,. recurrence at 1m PE sensitive further recurrence at 2m	1m	Dialysis	Ongoing	Y	NA	Remission 228	10m
Alachkar et al. (2012)	NI	1st Tx recurrence 2m, graft loss	32y 2nd Tx recurrence at 10 weeks. PE resistant	∼2w	Dialysis	8m	Y	Recurrence 5m after Ecu stopped – pneumonia^c^	Ecu restarted but graft loss after ATN. Ecu discontinued	NA
Ardissino et al. Zuber et al. (2012b)	*CFH*	No	6y 1st Tx, recurrence 2m, PE resistant	2d	442	Ongoing	Y	NA	Remission 48	25m
Zuber et al. (2012b)	*CFH* S1191L V1197A	1st Tx recurrence, graft loss	23 y 2nd Tx recurrence 3d. PE resistant	3d	627	Ongoing	Y	NA	Remission 65	17m
Zuber et al. (2012b)	*CFH/CFHR1* hybrid	4 previous Tx – 2 due to recurrence, 2 due to thrombosis	27y 5th Tx, recurrence 3d PE partially sensitive	1m	237	Ongoing	Y	Fresh TMA lesions at 3 months^d^	Remission 204	12m
Zuber et al. (2012b)	Anti FH Ab ΔCFHR1/3	4 previous Tx, 3 due to recurrence	41y 5th Tx, recurrence 5y^e^ PE partially sensitive	3m	89	Ongoing	Y	NA	Remission 80	9m
Guentin et al. Zuber et al. (2012b)	*CFI* G101R	1st Tx, recurrence graft loss	42y 2nd Tx, 15m of prophylactic PE, 8m taper recurrence at 13m after stopping. PE resistant	9w	190	Ongoing	Y	Relapse following delay prior to 5th infusion	Remission 156	4.5m
Heyne Zuber et al. (2012b)	NI	1st Tx recurrence graft loss	43y 2nd Tx, recurrence 8d no PE	1d	176	8m	Y	Relapse 3 months after stopping influenza vaccine triggered	Remission 123	14m

NI: not identified; ND: not documented; Tx: renal transplant; Ecu: Eculizumab; SCr: serum creatinine.

a In remission already.

b aHUS recurrence with AKI when injection delayed by 6–8 days.

c Following Ecu resumption, patient had endovascular procedure leading to severe ATN and subsequent graft loss.

d Biopsy of transplant allograft in response to falling haptoglobin level.

e Graft biopsy due to slight decrease in renal function disclosed fresh TMA lesions.

**Table 3:** Summary of 10 patients who received Eculizumab as pre-emptive treatment for aHUS in renal transplantation.

Reference	Mutation	Previous transplants	Age (y) at current Tx	Type of donor	Received PE^a^	When Eculizumab started^a^	Outcome SCr (μmol/L)	Follow Up
Zimmerhackl et al. (2010)	*CFH* W1183C	No	10	DD	9 PE day 0 to day 9	Day 10	No recurrence 44	2y 1m
Weitz et al. (2011)	*CFH* E1198X	No	7	DD	No	Day-21^b^	No recurrence normal SCr	7m
Nester et al. (2011)	*CFH/CFHR1* hybrid	No	12	LNR	2 PE day-7 and day-1	Day 7 and -1	No recurrence 80	4m
Krid et al. (2012)	*CFH/CFHR1* hybrid	No	7.5	DD	No	At time of transplant	No recurrence normal SCr	16m
Rondeau et al. Zuber et al. (2012b)	Complex recombination between *CFH* and *CFHR1*	No	18	DD	6PE day 0 to day 5	Day 5	No recurrence	14m
Lahoche Zuber et al. (2012b)	*C3* R161W	No	6.4	DD	No	At time of transplant	No recurrence 44	4.5m
Krid and Niaudet Zuber et al. (2012b)	*CFH* Q1137X	No	9	DD	No	At time of transplant	No recurrence 58	2m
Hourmant Zuber et al. (2012b)	*CFH* S1191L	1st Tx, recurrence.	18	DD	1PE day 0	At time of transplant^c^	Graft loss^d^	NA
Zuber and Legendre Zuber et al. (2012b)	*CFH* Y1177C	1st and 2nd Tx recurrence	41	DD	No	At time of transplant	No recurrence 176	2m
Xie et al. (2012)	*CFH* E625X	No	30	LNR	2PE day-7 and day-1	Day-7 and day-1	No recurrence ∼77	1y

NA: not applicable; DD: deceased donor; LNR: live non-related donor; SCr: serum creatinine; Ecu: Eculizumab.

a Timings in days in relation to day of renal transplantation.

b Received weekly doses until transplantation.

c Discontinued after nephrectomy.

d Early arterial thrombosis at day 1, nephrectomy day 3.

### 9.4 Eculizumab and the treatment of aHUS in native kidneys

There are currently 19 reported cases of the use of Eculizumab in aHUS in native kidneys (Table 1). Over half were in children and the oldest patient was 50 years. While the clinical course of disease leading to the commencement of Eculizumab was variable, response was overwhelmingly positive, albeit with the caveat of publication bias towards successful cases. Plasma exchange was attempted in 17/19 patients prior to the use of Eculizumab. Most (13/19) patients had severe renal failure or had already commenced renal replacement therapy. The time from diagnosis until use of Eculizumab was variable. There were 6 patients who received Eculizumab early in their clinical course (<2 weeks). Five were on dialysis and four patients had some plasma exchange. The fifth patient described by (Ohanian et al., 2011a, Ohanian et al., 2011b) had no plasma exchange – concerns regarding her neurological status prompted the first-line use of Eculizumab. In a further paediatric case Eculizumab was used as first line treatment successfully (Gilbert et al., 2013).

The general outcome was of an improvement in TMA and most patients had improvement in their renal function, including many of those already on dialysis. Povey et al. (2013) reported a case of a 21-year old unsuccessfully treated with PE who responded to Eculizumab and regained almost normal renal function despite over 3 months of dialysis requirement. Three patients did not recover renal function. Mache et al. (2009) describe resolution of the TMA and some renal recovery following a single dose of Eculizumab, given 3 months after diagnosis of aHUS. Following a relapse 2 weeks later, 3 more doses of Eculizumab were given. This again corrected the TMA but the patient had developed ESRD and Eculizumab was stopped. Kose et al. (2010) report initial control of TMA and renal improvement following a single dose of Eculizumab but a relapse after 2 months led to ESRD. Garjau et al. (2012) report failure to recover renal function following initial treatment with PE and commencing treatment with Eculizumab 90 days after diagnosis for a total of 27 weeks. The TMA, however, did improve.

Heinen et al. (2013) and Lapeyraque et al. (2011) report treatment following a long relapsing-remitting course highly dependent on PE. The period of plasma treatments had spanned 11 and 5 years respectively. In the former case, a switch to Eculizumab maintained TMA remission (though the patient already had established ESRD without improvement). In the latter case, PE resistant TMA had developed and Eculizumab successfully corrected TMA with improvement in renal function.

### 9.5 Eculizumab in recurrent aHUS in renal transplantation

There are currently 15 reported cases of the use of Eculizumab for the treatment of recurrence of aHUS in renal allografts (Table 2). Nine patients had documented mutations which are considered high risk for recurrence. The experience in early childhood is limited – with only one child (6 years). The others ranged from 15 years to 43 years. Eight patients received Eculizumab within one month of disease recurrence. The longest time from recurrence to commencement of Eculizumab was 15 months. All, but one patient, received PE as part the treatment for recurrence of aHUS (prior to receiving Eculizumab), including five patients who were on pre-emptive PE strategies. All patients responded to Eculizumab, irrespective of their clinical course leading up to the use of Eculizumab. Eight patients have remained on Eculizumab and have been in remission throughout.

Nurnberger et al. (2009) report giving a single dose of Eculizumab resulting in early remission although the patient subsequently lost their graft at 21 months – without biopsy proven TMA. In a separate case, a single dose initially controlled disease but aHUS recurred at 11 months necessitating reintroduction of Eculizumab (Larrea et al., 2010). Subsequent humoral rejection ultimately led to graft loss.

In two reported cases (Alachkar et al., 2012, Zuber et al., 2012b) Eculizumab initially controlled aHUS recurrence in the transplant and treatment was stopped at 8 months. One patient developed pneumonia 5 months after discontinuation of Eculizumab leading to a relapse of aHUS and worsening renal function. Eculizumab was recommenced resulting in improvement in TMA and renal function. Following an endovascular procedure, this patient developed severe acute tubular necrosis with poor recovery. Progressive renal failure followed leading to graft loss after 2 years. Eculizumab was only discontinued at this time-point – there was no evidence of TMA throughout this time. The other patient had a relapse of aHUS 3 months after discontinuation of Eculizumab, triggered by influenza vaccination. Eculizumab was restarted and remission has been maintained. In two further cases (Chatelet et al., 2010, Zuber et al., 2012b) minor relapses followed delays in dosing. Zuber and Legendre (Zuber et al., 2012b) report one patient who had a low haptoglobin level between 6 and 12 weeks of starting Eculizumab. Renal biopsy was therefore undertaken demonstrating fresh TMA lesions despite ongoing Eculizumab treatment. CH50 was below the level of detection and renal allograft function was stable.

### 9.6 Pre-emptive use of Eculizumab in renal transplantation

With the high aHUS recurrence rate in renal allografts in individuals with complement mutations, pre-emptive Eculizumab has been given in ten cases (Table 3). All patients had a complement mutation that is associated with a high risk of disease recurrence. Two patients had PE prior to renal transplantation and continued until days 5 and 10 respectively before receiving Eculizumab. Three patients had PE followed by Eculizumab pre-operatively and the remaining five patients received only Eculizumab (no PE) at the time of transplantation. Most (9/10) patients remain in disease remission (and still receiving Eculizumab) with excellent graft function with follow-up ranging from 2 months to over 2 years. Only 1 patient had graft loss and this was due to arterial thrombosis in a patient with S1191L mutation in *CFH*.

### 9.7 Prospective trials

Although the results of the prospective trials have as yet, only been reported in meeting abstracts (Greenbaum et al., 2011, Greenbaum, 2011, Legendre et al., 2010, Licht, 2011, Licht et al., 2011, Muus et al., 2010) from the information available, Eculizumab seems highly effective, with ∼85% of patients becoming disease free in both plasma-resistant and plasma-dependent aHUS (reviewed Zuber et al., 2011). It has been suggested that Eculizumab achieves better control of disease as witnessed by improvement in renal function following switching from PE and in rescuing plasma resistant individuals (Zuber et al., 2011). It should be noted however that a randomised trial of Eculizumab against PE was not and is unlikely to be performed.

### 9.8 Adverse effects of Eculizumab

There were few side effects documented in the review of the case reports. Adverse effects were reported in preliminary data from the prospective open-label clinical trials of Eculizumab in aHUS. Campistol et al. (2013) summarise this data and note 4 reports (out of 37 patients) of serious adverse effects (peritonitis, influenza infections, venous disorder and severe hypertension). The use of Eculizumab has been approved for use in PNH following a successful clinical trial. In this study there were only 4 serious adverse effects (compared to 9 in placebo). These were exacerbation of PNH, renal colic, lumbar- or sacral-disc prolapsed and α-haemolytic streptococcal bacteraemia (Hillmen et al., 2006). Vaccination against *Streptococcus pneumonia* and *Haemophilus influenza B* in children treated with Eculizumab has been recommended (Zuber et al., 2011).

### 9.9 Risk of meningococcal infection

It is well recognised that loss of ability to form membrane attack complex is associated with infection due to *Neisseria meningitidis*. Patients receiving Eculizumab should therefore receive the tetravalent vaccine (A,C,Y,W135). Because the vaccine does not cover the most prevalent serogroup in Europe – serogroup B (Bouts et al., 2011) we recommend ongoing prophylactic penicillin in Eculizumab treated patients.

### 9.10 When to start Eculizumab?

We believe that there is a clear role for the use of Eculizumab in the treatment of aHUS. Despite this PE should remain the initial treatment in adults until ADAMTS13 deficiency can be excluded. In paediatric cases where the risks and difficulties of administrating PE are high, it has been suggested that Eculizumab be used first line. The success of such a strategy depends on the differentiation of aHUS from TTP and the authors are aware of at least one case where congenital ADAMTS13 deficiency would have been inappropriately treated with Eculizumab (based on platelet count, creatinine and clinical presentation).

Given the apparent superior efficacy of Eculizumab over PE (Zuber et al., 2011), the authors believe that once ADAMTS13 deficiency has been excluded all patients should be managed with Eculizumab. While genetic testing should be performed in all patients with aHUS, it need not delay treatment with Eculizumab. Clearly, the prohibitive cost of Eculizumab will mean that in many parts of the World, PE remains the only available treatment.

In patients undergoing transplantation with high risk mutations (e.g. *CFH*, *C3* and *CFB*), the unavoidable ischaemia–reperfusion injury induced complement activation make pre-emptive Eculizumab the treatment of choice in our opinion.

The penetrance rate in families with mutations is low and as such there will be many family members potentially at risk of disease when exposed to a triggering stimuli. Currently in family members with known mutations our strategy has been to advise monitoring at times of high risk (e.g. pregnancy, respiratory tract infections, etc.).

### 9.11 When to stop Eculizumab?

Ultimately, the aim is to balance the risk of Eculizumab (see side-effects) against effective treatment and prevention of recurrence of TMA and the organ-specific damage associated with it. Early cessation of Eculizumab has given rise to recurrence of disease. This is perhaps unsurprising with a terminal cascade blocking agent, as the C3 convertase will initially still be active on the glomerular vasculature. As such, a sustained course of treatment should be given to maintain inhibition of complement activity. The reported experiences suggest that even in individuals who have presented requiring dialysis, Eculizumab treatment can control disease eventually leading to recovery of renal function after several months. It is tempting to treat every patient with long-term Eculizumab, but the cost and unknown long-term effects of this treatment need to be considered. There are certain groups of individuals where withdrawal of Eculizumab is likely to be successful (e.g. *MCP* mutations). Such a strategy should be performed under the auspices of a clinical trial with careful monitoring.

Likewise in individuals treated with Eculizumab where genetic analysis subsequently reveals mutations in *DGKɛ*, treatment can be stopped. This pathway does not seem to involve the complement system and as such complement modulatory therapy is unlikely to be efficacious. Indeed, one patient with mutations in *DGK ɛ* developed aHUS while on treatment with Eculizumab (Lemaire et al., 2013).

In individuals where TMA has been controlled but renal function has not recovered Eculizumab has usually been discontinued. There are, however, rare reports of severe extra-renal manifestations such as cerebral artery stenoses (Kaplan et al., 2000, Loirat et al., 2010, Vergouwen et al., 2008) which have been attributed to ongoing complement activation. The experience is currently too limited to recommend routine ongoing treatment with Eculizumab in individuals on long term dialysis.

### 9.12 Single nucleotide polymorphisms in C5

It had been noted in a Japanese PNH population that certain individuals did not respond to Eculizumab despite adequate serum levels (Nishimura et al., 2012). In vitro assays of these non-responders’ serum demonstrated an inability of Eculizumab to prevent haemolysis. An antibody against a different epitope on C5 did, however, block haemolysis. Genetic analysis of these non-responders revealed a SNP in C5 (p.R885H) which predicted a poor response to Eculizumab (Nishimura et al., 2012). This SNP was present in around 2% of a Japanese population. In individuals with aHUS who fail to respond to Eculizumab, PE should be re-instituted and genetic analysis of *C5* performed.

### 9.13 Long term treatment with Eculizumab

While the effect of Eculizumab in preventing ESRD in aHUS seems inescapable, long term follow up is still awaited. It is interesting to note that an aHUS patient who received the drug seems to have deposited the Eculizumab in glomeruli and tubular basement membranes (Herlitz et al., 2012). This has also been reported in individuals receiving treatment for C3 glomerulopathies and DDD where Eculizumab was deposited in a similar distribution and was said to resemble monoclonal immunoglobulin deposition disease (Herlitz et al., 2012). Similarly, as would be expected in a terminal pathway blocking therapy, these individuals continued to deposit C3 in the kidney (Herlitz et al., 2012). While the outcome of these effects remains to be established, the longer term evidence from PNH treatment would not suggest worsening of kidney function or development of proteinuria (Hillmen et al., 2010).

## 10 Summary

In the last 15 years the elucidation of the role of complement in pathogenesis of aHUS has seen a sea change in the management of disease. The complement inhibitor, Eculizumab, has now been demonstrated to be effective in controlling aHUS, however, its prohibitive cost will mean that in many parts of the World, PE will remain the only treatment option.

## Added in press

The prospective trial of Eculizumab in aHUS demonstrating efficacy has now been published (Legendre et al., 2013).
